# Successful pregnancy after breast cancer therapy: dream or reality?

**DOI:** 10.1186/1477-7800-6-7

**Published:** 2009-03-02

**Authors:** Konstantinos Kontzoglou, Michael Stamatakos, Sofia Tsaknaki, Helen Goga, Alkiviades Kostakis, Michael Safioleas

**Affiliations:** 1 2nd Department of Propedeutic Surgery, Medical School, University of Athens, Laiko General Hospital, Athens, Greece; 2 1st Department of Pathology, Medical School, University of Athens, Laiko General Hospital, Athens, Greece; 3 4th Department of Surgery, Medical School, University of Athens, ATTIKON General Hospital, Athens, Greece

## Abstract

**Background:**

Nowadays, more breast cancer patients want to have children after the diagnosis of cancer. The purpose of this study is to review the possibility and risks of giving birth among women with breast cancer previously treated by chemotherapy.

**Case presentation:**

Two young women aged 28 and 34 respectively, were treated in our clinic for breast cancer, the first (negative hormonal receptors) by surgery, chemotherapy and radiotherapy and the second (positive hormonal receptors) by surgery, radiotherapy and tamoxifen. They both became pregnant, 1 and 8 years after completion of the therapy respectively.

**Results:**

Laboratory testing during pregnancy was negative in both cases and after an uneventful course each woman gave birth to a perfectly healthy child. The first patient breastfed her baby for three months, while the second one did not breastfeed her baby at all.

**Conclusion:**

Women undergoing chemotherapy for breast cancer can maintain their fertility and get pregnant. Previous chemotherapy for breast cancer does not present any supplementary risks for the child's mental or physical health.

## Introduction

Women, nowadays, delay their childbirth to the 30ies. Therefore, more breast cancer patients have not completed their family planning and desire to have children after the diagnosis of breast cancer, though, there are no valid treatment options for preserving ovarian function after chemotherapy. Many authors believe that there is no increased risk for relapse if a breast cancer patient becomes pregnant. However, the timing of pregnancy has not yet been fixed. It depends on the factors of prognosis, the age, the personal situation and personal preferences [1].

## Case presentation 1

A twenty-eight year old female patient presented to the breast clinic with a palpable painful mass on the upper lateral quadrant of the right breast, measuring 3 × 4 cm^2^. Upon further questioning, menarche occurred at the age of thirteen and it was stated that her menstrual cycle was regular. The patient denied use of contraceptive pills. No birth, miscarriage or abortion is mentioned. The patient was a smoker. There were no inherited diseases or family history of cancer.

A biopsy under general anesthesia was performed, which was positive; therefore an extended tumorectomy and radical lymph node dissection were performed. The histological testing showed invasive ductal carcinoma of myeloid type, grade III with 4/12 of the axillary lymph glands involved, as well as coexistence of carcinomatic emboli to the lymphatic vessels. The surgical margins were clear of neoplasmatic cells. The tumor was negative to PR and ER: 5% progesterone receptors, 10% estrogen receptors, 0% p53, c-erbB-2: score +3, 60%, ki 67. The patient received chemotherapy (clitaxel, epirubicin, paclitaxel, cyclophosphamide, methotrexate) for four months and radiotherapy for one month.

A year after the accomplishment of the adjuvant treatments she got pregnant and was under supervision thereafter. The pregnancy was uneventful and a healthy child with no health problems (normal physical and mental development) was born, by natural labor.

The patient breastfed her baby for three months. Routine follow-ups were performed every month, included ultra sound (U/S) cancer ratings, with normal findings.

Four years post-labor, the patient remains free of disease.

## Case presentation 2

A thirty-four-year old female patient presented to the breast clinic with a palpable painless mass on the upper lateral quadrant of the right breast. Menarche occurred at the age of fourteen, as well as regular menstrual cycle was stated. No contraceptive pills use is mentioned. It was also mentioned that she has had children, no miscarriage or abortion. The patient was a smoker. Her family medical record was also free of inherited diseases or cancer.

The patient was submitted to immediate biopsy under general anesthesia (which was positive). Therefore, a broad resection of the quadrant of the right breast with respective lymphadenic care was performed. The histological testing showed ductal invasive carcinoma grade II with 2/13 of the axillary lymph glands involved. The surgical limits were clear of neoplasmatic cells. The tumor was positive to ER and PR: 90% progesterone receptors, 80% estrogen, 0% p53, c-erbB-2: score+1, 15% ki 67. The patient received radiotherapy for two months (40 sessions) and she was given anti-estrogen (Tamoxifen) for two and a half years, which was discontinued due to the occurrence of ovary cysts and menstrual disturbances.

Eight years after the accomplishment of the adjuvant treatments she got pregnant under supervision. The pregnancy was uneventful and a healthy child was born via a caesarian incision. The patient did not breastfeed her baby. Routine follow-ups were performed every month, included ultra sound (U/S) and cancer indexes testing, which both proved to be negative throughout the whole period.

Thus, twelve years post-mastectomy, during the check-up performed with bone CT scanning, deposits were found on the bones and the ultra-sound showed liver metastasis, measuring approximately 3 cm. The patient received chemotherapy (Fluorouracil, Adriamycin, Cyclophosphamide) for six months. After the six-month period she continued Tbs treatment (Noveldin) for three months.

Till present, according to the C/T scans, the metastasis in the liver measures 1 to 1,5 cm and the patient continues her treatment with Zometan for bone deposits.

Generally patients were monthly checked by clinical evaluation, cancer indices rating (CEA, Ca 125, Ca 153) and breast U/S during pregnancy and immediately after birth; Thereafter, they should be checked every three-six-nine-twelve months and then yearly with the addition of CT scanning of thorax, CT of upper and lower abdomen and bone scanning.

## Discussion

Cancer is the leading cause of death among women, aged 25–55. Among the new cases of breast cancer diagnosed, approximately 10% are women younger than 44 years of age.

Young breast cancer survivors and their spouses may face a difficult dilemma regarding their wish to have children. As a result of new developments in breast cancer treatment, more women are cured or remain in long-term remission after being diagnosed with breast cancer. This, along with the growing trend in western societies for women to delay childbirth, results in an increasing number of young breast cancer survivors, who have not yet started or completed their childbearing years [2-4].

The common recommendation for women with early stages of breast cancer is to wait at least 6 months up to two years after the end of their treatment before trying to become pregnant, because most of the recurrences of cancer occur during this period of time [5].

It has been shown that the majority of women, younger than 35 years of age experience only temporary amenorrhea due to chemotherapy and can maintain fertility [6]. The medical literature has not demonstrated a survival disadvantage [3,5-7], or a higher proportion of distant metastasis [8], in women who have given birth after breast cancer compared to women who have not given birth after breast cancer. In addition, although chemotherapy has been shown to result in increased miscarriage frequency [7], children born to mothers who have received chemotherapy are not at a higher risk for congenital defects compared to the general population [9]. Nevertheless, in their latest review on the subject, Upponi SS et al [10] concluded that "the effect of subsequent pregnancy on patients who have had breast cancer with regard to local recurrence, distant metastasis and survival remains debatable, due to sampling and methodological limitations (eg, Retrospective studies)". There is concern with subsequent pregnancy after breast cancer treatment that a high concentration of estrogen in the blood during pregnancy may contribute to breast cancer recurrence [5,9]. In addition, it was found that pregnancy is a risk factor of development of breast cancer among carriers of BRCA1 and BRCA2 genetic mutations [11]. It has been assumed that the immuno-suppressant and hormonal effects of pregnancy so close to diagnosis and during treatment may have a significant deleterious effect. Consistent with this theory, Nugent and O'Connell reported a worse outcome for women with early subsequent pregnancy. Other authors have refused this, showing no worse outcome with subsequent pregnancy. In fact, women who have a subsequent pregnancy have equivalent or, possibly, better survival matched for stage. Although this suggests that subsequent pregnancy may provide a survival benefit, there may be observational bias involved, with only a selected group of women, who feel healthy give birth and those who are affected by the disease do not: a "healthy mother" effect [12,13]. There are many aspects that need to be considered when pregnancy (planned or not) is a possibility subsequent to a diagnosis of breast cancer. These include social, psychological, economic, and biological issues in a woman who has a potentially limited lifespan. The woman (and her family) will have concerns about whether she will remain fertile following treatment, what influence a subsequent pregnancy may have on recurrence of breast cancer and its consequences, and whether the child may inherit a familial tendency towards breast cancer [4].

Recently, at the ninth St Gallen expert consensus meeting in January 2005, the experts suggested that women with breast cancer should be offered chemotherapy for endocrine non-responsive disease and endocrine therapy as the primary therapy for endocrine responsive disease, adding chemotherapy for some intermediate- and all high risk groups in this category [14]. Just as limited surgery, chemotherapy and endocrine therapy allows conservation of the breast and unaffected lymph nodes and limited radiation therapy is being studied, thus appropriate adjuvant systemic therapy involves choosing treatments tailored to individual patients according to assessment of endocrine responsiveness [14]. Concerning the matter of metastases, the hypothesis that a subset of tumor cells ('tumor stem cells') are responsible for invasion and metastases, while other tumor cells are not tumorigenic, is supported by experiments on human breast cancer cells in immunocompromised mice [15,16]. A specific gene expression signature seems to characterize these cells and even specifically be associated with an organ specific metastatic potential [17].

Such stem cells might become a major target for tailored therapies.

Prognosis of DCIS, which both of our patients presented, is associated with size, grade, distance to resection margins, degree of ER expression and age. Tamoxifen reduces recurrence of receptor positive DCIS, but shows little evidence of benefit in receptor negative disease [18]. Radiotherapy reduces in situ and invasive recurrence but is sometimes omitted, especially for small low-grade lesions [19,20], as the second patient was treated. Moreover, steroid hormone receptors (Era, PgR), according to new data, are overwhelmingly indicative of endocrine responsiveness, though not all tumors expressing detectable hormone receptors will have a clinically useful response, like in the case of the second patient. Tumors completely lacking such receptors were found to be particularly sensitive to preoperative cytotoxic agents, but despite a pathological complete remission rate exceeding 30%, survival of patients with this phenotype was shorter than for patients with receptor-positive tumors who obtained pathological complete remission significantly less frequently [21].

Exclusion of multifocal disease by MRI may improve selection for conservative surgery.

The second patient was not submitted to MRI control, but she presented an interval free of disease. Conservation of the nipple-areola complex during mastectomy, with immediate breast reconstruction, while well accepted by patients [22], requires careful patient selection and patient information on limited long-term experience.

Breast radiation reduces relapses in the breast and chest wall. On the average, for patients at high risk of local relapse, radiation therapy was also shown to improve survival [23]. Radiation therapy is clearly indicated after breast conserving surgery including a boost in younger patients. It remains uncertain whether radiation therapy may be omitted for women above the age of 70 years with clear margins who also receive adjuvant endocrine therapy [24]. The aforementioned are proved through the cases of our two patients.

The Columbia study was updated at a median of 20 years and concluded that for patients with high-risk breast cancer radiation therapy and adjuvant chemotherapy yielded better survival than chemotherapy alone [25]. This fact is greatly proved in case one, where combined therapy was applied, while in the second case it raised the negative effects of its application.

For premenopausal women with endocrine responsive disease, combined ovarian function suppression (goserelin) and tamoxifen appeared to be as effective as CMF chemotherapy [26,27]. The addition of tamoxifen after chemotherapy reduced relapse in premenopausal women with endocrine-responsive tumors but the same study found a detrimental impact of tamoxifen on disease-free survival in endocrine non-responsive disease [28]. Thus, correctly the first woman was not given tamoxifen, while no chemotherapy was given in the second case, which proved to lead to bad results.

Very young patients have a worse prognosis compared to older premenopausal women presenting with otherwise similar cancer [29,30]. Three high priority trials are specifically investigating ovarian function suppression (SOFT), an aromatase inhibitor (TEXT) and the need for chemotherapy (PERCHE) in adjuvant therapy for endocrine responsive breast cancer in young women [26,27].

Although p53 protein accumulation and gene mutation were implicated in resistance to chemotherapy, the clinical value of these findings remains controversial [31,32].

Similarly, multi-gene assay (eg, Oncotype DX for 16 genes) by RT-PCR was found to be useful for defining a group of patients with estrogen receptor positive disease who would benefit from chemotherapy added to tamoxifen [33], but further prospective validation of this method is required.

Treatment choice depends upon potential endocrine responsiveness although most studies have used chemotherapy, frequently containing anthracyclines and taxanes. In a small randomized study (42 patients) neo-adjuvant trastuzumab plus chemotherapy yielded a higher pathologic complete response rate than chemotherapy alone for patients with HER2-positive disease [34]. These data require confirmation.

Endocrine neoadjuvant therapy has also been successful [35,36]. Remaining questions include chemotherapy combined with endocrine treatments (eg, Aromatase inhibitors) and duration of systemic therapy for best timing of surgery [21].

## Conclusion

Healthy pregnancy can occur even after chemotherapy with no consequences for the fetus or even later for the child. These patients are of remarkable interest.

Even if there are various studies suggesting controversial results, concerning the hormonal receptors and the associated risk of tumorigenesis among young women, in cases of hormone-dependent cancer, it seems that hormonal receptors have the most important role regarding the patients outcome [14].

In conclusion, the possibility of recurrence of the disease remains a constant life long hazard, which seems to be related to the specific characteristics of the tumor and mainly, to hormonodependence.

## Abbreviations

PR: Progesterone receptors; ER: Estrogen receptors; p53: protein 53; C-erb: Avian erythroblastosis oncogene B; Ki: proliferation index; U/S: Ultrasonography; CT: Computed tomography; CEA: Carcinoembryonic antigen; CA-125: Cancer antigen 125; CA 15-3: Cancer antigen 15-3; BRCA 1: gene for breast cancer 1; BRCA 2: gene for breast cancer 2; DCIS: Ductal carcinoma in situ; ERa: Estrogen-related receptor; PgR: progesterone receptor gene; MRI: Magnetic resonance imaging; CMF: Cyclophosphamide-methotrexate-5-fluorouracil; SOFT: Suppression of Ovarian Function Trial; TEXT: Tamoxifen and EXemestane Trial; PERCHE: Premenopausal Endocrine Responsive CHEmotherapy; RT-PCR: Real time polymerase chain reaction; HER2: Human Epidermal growth factor Receptor 2.

## Consent

Written informed consent was obtained from the patients for publication of this case report and accompanying images. A copy of the written consent is available for review by the Editor-in-Chief of this journal.

## Competing interests

The authors declare that they have no competing interests.

## Authors' contributions

KK is the surgeon, who performed the operation and edited a part of the manuscript. MS is the surgeon who performed the operation and prepared the draft. ST did the literature search and the revision of bibliography. HG is the oncologist, who was responsible for the treatment of cancer patients. AK is the surgeon who performed the operation. MS is the surgeon, who performed the operation and edited a part of the manuscript.
